# Microtubule-Dependent Trafficking of Alphaherpesviruses in the Nervous System: The Ins and Outs

**DOI:** 10.3390/v11121165

**Published:** 2019-12-17

**Authors:** Drishya Diwaker, Duncan W. Wilson

**Affiliations:** 1 Department of Developmental and Molecular Biology, Albert Einstein College of Medicine, 1300 Morris Park Avenue, Bronx, NY 10461, USA; drishya.diwaker@einsteinmed.org; 2 Dominick P. Purpura Department of Neuroscience, Albert Einstein College of Medicine, 1300 Morris Park Avenue, Bronx, NY 10461, USA

**Keywords:** herpes simplex virus, pseudorabies virus, neurons, anterograde axonal transport, retrograde axonal transport, microtubules, motors

## Abstract

The *Alphaherpesvirinae* include the neurotropic pathogens herpes simplex virus and varicella zoster virus of humans and pseudorabies virus of swine. These viruses establish lifelong latency in the nuclei of peripheral ganglia, but utilize the peripheral tissues those neurons innervate for productive replication, spread, and transmission. Delivery of virions from replicative pools to the sites of latency requires microtubule-directed retrograde axonal transport from the nerve terminus to the cell body of the sensory neuron. As a corollary, during reactivation newly assembled virions must travel along axonal microtubules in the anterograde direction to return to the nerve terminus and infect peripheral tissues, completing the cycle. Neurotropic alphaherpesviruses can therefore exploit neuronal microtubules and motors for long distance axonal transport, and alternate between periods of sustained plus end- and minus end-directed motion at different stages of their infectious cycle. This review summarizes our current understanding of the molecular details by which this is achieved.

## 1. *Alphaherpesvirinae* and the Road Travelled: An Overview

The *Herpesviridae* is a large family of structurally complex enveloped dsDNA viruses that establish lifelong latent infections, with periodic reactivation, in their hosts [1]. Family members exhibit a range of tissue tropisms and replication strategies, with the subfamily *Alphaherpesvirinae* including those that replicate in peripheral tissue then invade the nervous system to establish latency [1,2,3]. *Alphaherpesvirinae* of humans include herpes simplex virus types 1 and 2 (HSV-1 and HSV-2, of the genus *Simplexvirus*) and varicella zoster virus (VZV, of the genus *Varicellovirus*) [4,5]. Our understanding of how *Alphaherpesvirinae* invade neurons and exploit their microtubule (MT)-directed trafficking machinery has benefited from synergistic studies of these human pathogens and alphaherpesviruses of veterinary importance, most notably the swine *Varicellovirus* pseudorabies virus (PRV, suid herpesvirus 1) [2,3,6,7].

**Figure 1:**
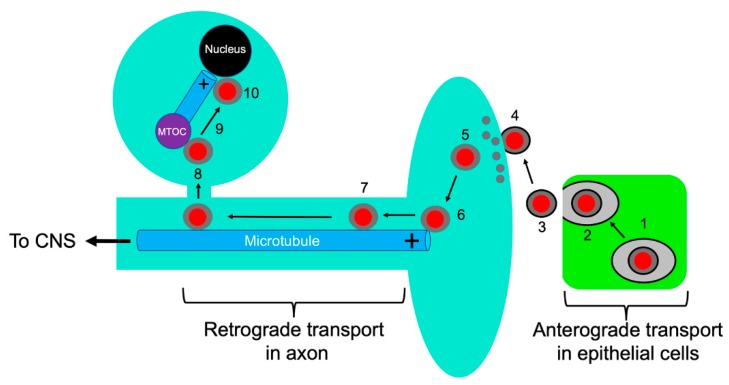
Alphaherpesvirus entry into neurons. Capsids are represented as red discs and the UL36p/UL37p inner tegument as a gray capsid-bound layer. Microtubules are blue rods with the + end indicated. Virions replicate and assemble in infected epithelial cells (green) (1) and exocytosis (2) releases infectious enveloped particles (3) that fuse at the surface of adjacent sensory neurons (4). Tegument partially disassembles (grey discs) (5), and the capsid with associated inner tegument attaches to the plus end of axonal microtubules (6). The tegument-bound capsid then recruits dynein/dynactin and proceeds by MT-directed retrograde axonal transport (7), eventually reaching the MTOC (purple disc) (8). The capsid then switches to an anterograde trafficking mode (9) to deliver the viral genome to the cell nucleus (10).

Transmission of neurotropic herpesviruses between individuals is commonly via delivery of infectious virions to peripheral locations such as exposed epithelial cells, rather than directly to neurons where life-long latent infection will be established. Consequently, the first task a newly transmitted alphaherpesvirus faces is to establish productive replication in somatic cells at the site of infection, generating an inoculum of viral particles for subsequent delivery to neurons [2,3]. This commonly occurs in mucosal epithelia such as the oral and anogenital mucosa for HSV-1 [4,5] and nasal and oropharyngeal mucosa for PRV [7]. Following replication in these tissues, viral particles are released and infect the termini of adjacent sensory neurons (Figure 1) [8,9,10]. They then travel by MT-directed retrograde traffic along the axon to the neuronal cell body. The viral genome is ultimately delivered to the nucleus and persists as a circular dsDNA episome during ensuing latency in the trigeminal ganglia (TG) of humans (HSV-1) and swine (PRV) [3,4,7]. Periodic reactivation [5,7] results in viral DNA replication, gene expression, and assembly of progeny viral particles that leave the nucleus and travel down axonal MTs in the anterograde direction to re-infect peripheral epithelial tissues [2,3,10]. The *Alphaherpesvirinae* must therefore ensure sustained retrograde axonal transport from the nerve terminal to the cell body to establish latency, and efficient anterograde delivery of progeny virions from the cell body down the axon following reactivation. The focus of this review is upon the molecular mechanisms by which alphaherpesviruses engage molecular motors and neuronal MTs to accomplish these goals [2,5].

## 2. Structure of the Trafficking Alphaherpesvirus Particle

**Figure 2:**
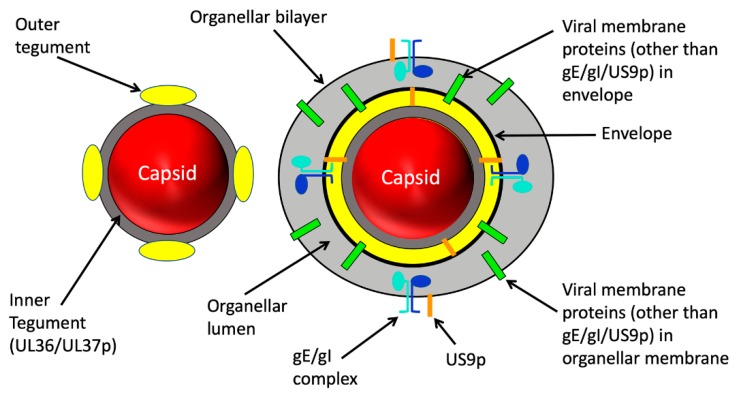
Structure of alphaherpesvirus particles that utilize MTs during assembly and traffic. Left: The naked capsid. After assembly and packaging in the nucleus the capsid (red sphere) is delivered to the cytoplasm. The inner tegument proteins UL36p and UL37p (dark grey layer) provides the foundation for attachment of outer tegument subunits (yellow) prior to or during cytoplasmic envelopment (not shown). Right: the organelle-enclosed enveloped virion (OEV). Budding of the naked capsid into the lumen (light grey space) of a cytoplasmic organelle is accompanied by completion of the outer tegument (yellow) and generates a mature infectious enveloped virion within the organellar lumen. Multiple virally encoded membrane proteins (green bars) reside in the viral envelope and surrounding organellar membrane. For the purposes of this review three virally encoded membrane proteins are shown in detail: the gE/gI glycoprotein heterodimer (subunits shown in light blue and dark blue) and the US9p protein (orange bar). Note that US9p has essentially no lumenal domain. US9p and the gE/gI complex in the organellar bounding membrane project tails into the cell cytoplasm.

The complexity of alphaherpesvirus MT-dependent transport within the nervous system reflects the intricate structure of the virions and the fascinating interplay of capsids and cellular organelles during assembly and transport (Figure 2) [3,10,11]. Alphaherpesvirus particles are typically composed of about 40 structural proteins distributed between three distinct layers (Figure 2): a ~125 nm diameter icosahedral capsid containing a linear dsDNA genome encoding approximately 80 open reading frames, an envelope constructed from the lipid bilayer of the host cell containing multiple virally encoded membrane proteins, and a complex protein layer termed tegument that lies between capsid and envelope. The structure and composition of alphaherpesvirus particles and their pathway of assembly have recently been reviewed elsewhere [6,12,13,14,15].

**Figure 3:**
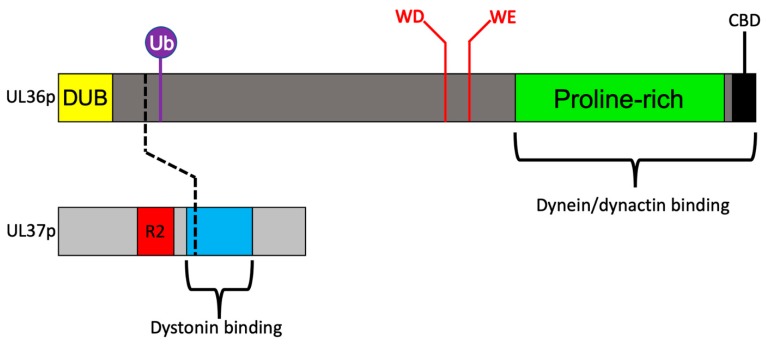
The UL36p and UL37p inner tegument proteins. Upper bar: The UL36p polypeptide. Regions discussed in this review are annotated as follows: DUB (Deubiquitinase) domain: yellow. Ub (Ubiquitin) attachment site: purple lollipop. WD/WE (tryptophan-acidic) motifs: red text. Proline-rich region: green. CBD (carboxy terminal capsid binding domain: black (additional capsid binding domains exist in UL36p but are not shown). Region sufficient for dynein/dynactin interaction is bracketed. Lower bar: UL37p polypeptide. R2 surface region: red. Site of dystonin binding: blue. Broken black line connects regions of UL36p and UL37p implicated in their association.

The tegument layer plays a central role in the interaction of neurotropic herpesviruses with cellular MTs during entry and exit from neurons. Tegument is a complex of more than a dozen distinct virally encoded proteins in addition to several host proteins [6,12,16,17]. The “inner” tegument (Figure 2) is largely composed of UL36p (also known as VP1/2) [12,18] and its binding partner UL37p [12,19] (Figure 3). With a mass of ~330 kDa, UL36p is the largest protein encoded by the *Herpesviridae*, and is anchored to the vertices of the icosahedral capsid [6,20] via its attachment to the capsid subunit UL25p [12]. UL36p in turn recruits to the capsid its inner tegument partner UL37p [21,22,23,24,25], a ~120 kDa protein with an amino terminal portion structurally similar to cellular multisubunit tethering complexes (MTCs) that facilitate docking of transport vesicles to target membranes during their intracellular trafficking [26,27] and a carboxy-terminal region with conformational flexibility to potentially accommodate multiple binding partners [19]. UL36p and UL37p perform multiple functions, including capsid envelopment in the cytoplasm (Section 5.3) [12,28,29,30], motor recruitment, and MT-directed trafficking (discussed in detail below) and recruitment of virally encoded polypeptides to the outer tegument [6,12,31,32]. The outer tegument proteins (Figure 2) exhibit a network of genetically and biochemically defined interactions with UL36p/UL37p [23,25,31,32], with each other, and with the inward facing (equivalent to cytoplasmically disposed) portions of membrane-embedded envelope proteins [6,12,33,34,35,36].

## 3. Viral Entry into Neurons, Capsid Attachment to Microtubules, and Retrograde Transport

### 3.1. Entry and Retrograde Transport, an Overview

Infectious viral particles generated by productive replication in epithelia (Section 1) attach to the plasma membrane at the termini of adjacent sensory neurons (Figure 1). Attachment triggers conformational changes in the interior of the viral particle that prime it for disassembly [6], and is followed by fusion between the viral envelope and target cell bilayer catalyzed by envelope glycoproteins gH/gL and gB [37,38]. Although HSV-1 can enter some cell types by endocytosis and fusion between the envelope and endosomal membrane [39], it is clear that HSV-1 entry into neurons occurs by fusion between the envelope and plasma membrane at the axon terminus [6,8,9,11,37]. This delivers the de-enveloped “naked” alphaherpesvirus capsid directly to the host cytoplasm (Figure 1), in contrast to the strategy adopted by the neuroinvasive viruses rabies [40] and polio [41], which enter the cell by endocytic uptake at the nerve terminal. Consequently, whereas rabies virus and poliovirus reside within the endosomal lumen and travel along the axon as passive cargo of a pre-existing organellar transport pathway [42], alphaherpesvirus capsids must recruit motors directly to their surface to ensure sustained retrograde transport [3,6]. Association with molecular motors is critical for delivery to and from the neuronal cell body; if restricted to passive diffusion alone, a particle the size of an HSV-1 capsid would require over two centuries to travel 1 cm through the crowded, protein-rich axonal cytoplasm [43].

Imaging of fluorescently tagged HSV-1 and PRV capsids during their retrograde traffic in axons of chick primary sensory dorsal root ganglia (DRG) revealed that capsids move at velocities between 0.5 and 5.0 μm/s, with averages between 2.1 and 2.6 μm/s [44], similar to dynein/dynactin-mediated transport of cellular cargo in speed and processivity [45]. HSV-1 capsid transport dynamics were indistinguishable from those of PRV and similar in chick DRGs, mouse primary sensory neurons, and cultured human neuroblastoma cells [44]. Together, these data imply substantial conservation of the machinery of retrograde capsid transport between alphaherpesvirus genera (varicellovirus and simplexvirus) and between the neurons of humans, rodents, and birds. Interestingly, retrograde capsid traffic (like that of normal cellular cargo) is punctuated by brief periods of anterograde transport [9,46], suggesting that even during retrograde motion the capsid is associated with both dynein and kinesin motors. The presence of multiple motors can greatly enhance the processivity of cargo transport, even when those motors act in opposed directions [47].

### 3.2. Induction of Host Protein Synthesis in the Axon Upon Alphaherpesvirus Infection

PRV infection at the nerve terminal of primary rat superior cervical ganglia (SCG) neurons stimulates local axonal protein synthesis that is detectable as early as 2 h post infection [48]. This new protein synthesis is important for subsequent viral transport since addition of cycloheximide one hour before infection resulted in a greater than 60% decrease in the numbers of retrograde trafficking PRV particles in the axon, despite having no effect upon the efficiency of viral entry at the nerve terminus [48]. Interestingly, induction of axonal protein synthesis is also seen following physical damage to axons [49,50], where localized translation of importin-β and the Ran GTPase effector RanBP1 [49,51] promotes assembly of an importin-α/importin-β/dynein retrograde injury-signaling complex that is thought to bind cargo and deliver it by retrograde traffic to the neuronal cell nucleus [49,51]. When injury was induced by cutting primary rat SCG axons to divide them into portions connected to the cell body (proximal axons) and portions completely disconnected (distal axons), and each segment infected, the number of retrograde trafficking PRV capsids was ~65% fewer in proximal segments than uncut control axons or fully detached distal axons [48]. These data were interpreted to suggest that MT-bound retrograde trafficking PRV capsids and axonal damage signaling complexes utilize similar transport mechanisms, and are competing for shared limiting cellular components [48]. High resolution mass spectrometry revealed that proteins newly synthesized in the axon upon PRV infection include those involved in cytoskeletal remodeling, intracellular trafficking, signaling, and energy metabolism [48]. Among the most prominently induced are Pafah1b1 (platelet activating factor acetyl hydrolase 1B)/Lis1 (lissencephaly type 1), Peripherin and Annexin A2, and siRNA knockdown of each of these proteins significantly reduced the numbers of PRV capsids undergoing retrograde traffic along the axon [48]. Lis1 participates in organelle and vesicle transport [52,53] including large-vesicle, high load dynein-mediated traffic in axons [54]. It binds directly to dynein’s motor domain and promotes either low affinity or high affinity interactions of dynein with MTs [55]. It has been proposed that Lis1 ensures weak dynein-MT binding to permit kinesin-driven anterograde delivery of dynein motors to the plus ends of MTs. Lis1 then switches dynein to a tight MT-binding mode to support cargo loading and efficient retrograde transport [55]. Alphaherpesviruses might therefore induce Lis1 synthesis to support anterograde delivery of dynein motors to their site of loading onto capsids, and/or stabilize the interaction of capsid-dynein complexes with MTs for retrograde traffic. Peripherin is a neuronal-type III intermediate filament protein that in addition to being induced by PRV infection also redistributes from a punctate to patchy axonal localization [48]. Its role in uninfected cells is unclear but it has been implicated in axonal guidance, axonal regeneration, and vesicular trafficking [56]. Annexin A2 is a calcium-modulated phospholipid binding protein that regulates lipid raft organization at sites where the plasma membrane contacts the actin cytoskeleton [57], and it also influences the axonal localization of tau [58]. Translational upregulation of Annexin A2 could therefore play a role in remodeling of cortical actin during viral entry into the cytoplasm or could modulate the stability of axonal MTs in preparation for retrograde traffic.

### 3.3. Recruitment of Dynein and Dynactin by the Inner Tegument Protein UL36p

During entry into the cytoplasm, the capsid leaves behind its envelope and envelope proteins at the cell surface, and most of the outer tegument proteins dissociate [44,59,60,61,62] (Figure 3). However, for both HSV-1 and PRV at least a subset of the inner tegument proteins UL36p and UL37p (Figure 3), and a serine/threonine kinase encoded by the viral US3 gene, remain attached and accompany the capsid during its journey along the axon [44,59,60,61,62]. Early studies in non-neuronal cells revealed that incoming HSV-1 capsids associate with the heavy and intermediate chains of dynein and the p150^Glued^ subunit of dynactin [63,64]. Dynein was observed to attach to the capsid vertices, in the vicinity of UL36p attachment sites [18,63], and several lines of evidence suggest it is the inner tegument that plays an important role in recruitment of the dynein/dynactin complex [63,64,65]. Using in vitro MT-dependent trafficking assays, the inner (but not outer) tegument proteins were found to be important for binding of dynein and dynactin to HSV-1 capsids [66] and for dynactin-dependent capsid trafficking [67]. Moreover, purified HSV-1 capsids with bound UL36p and UL37p could associate with dynein and dynactin in vitro but capsids lacking inner tegument, or in which inner tegument was obscured by outer tegument proteins, did not [68]. A direct role for UL36p in motor recruitment was demonstrated by coimmunoprecipitation of PRV UL36p with dynein and dynactin. Efficient coimmunoprecipitation required a proline-rich region in the carboxy-terminal portion of UL36p (Figure 3) which, when deleted, severely impaired capsid retrograde axonal transport and PRV virulence [69]. Strikingly, UL36p was sufficient to mediate retrograde transport of a surrogate cargo; when targeted to the surface of mitochondria in vivo it redistributed these organelles to the perinuclear region of cells in the absence of other viral proteins [69]. Taken together, these data suggest UL36p is the key effector recruiting dynein and dynactin to the capsid vertices in both HSV-1 and PRV (Figure 3). A number of other virally encoded proteins have been proposed to interact with dynein including the capsid subunit VP26 [70], but they are either not essential for efficient dynein-mediated retrograde capsid transport [65] or do not accompany the capsid during its retrograde journey (summarized in [2,11]).

Association of dynactin with the incoming alphaherpesvirus capsid [64,68,69] is likely to contribute to the processivity of dynein-mediated retrograde motion [66,71], but it may also mediate attachment of capsids to the plus ends of MTs by connecting them to the EB1/CLIP-170 (end-binding protein 1/cytoplasmic linker protein 170) complex [72,73]. EB1 is a +TIP (plus-end-tracking protein) that recognizes the growing ends of dynamic MTs and recruits other regulatory +TIPS to them [74,75]. Depletion of EB1, CLIP-170, or dynactin causes severe defects in retrograde transport of HSV-1 capsids in normal human dermal fibroblasts by disrupting capture at the plus ends of MTs, rather than reducing retrograde processivity [72]. The importance of EB1/CLIP-170/dynactin for plus-end capture of incoming alphaherpesvirus capsids by axonal MTs is not known, however EB1/EB3 (end-binding protein 3)-positive MTs are enriched in the distal axons of mouse DRGs, and an EB1/EB3-CLIP-170/dynactin-dependent process initiates the retrograde transport of mitochondria, early and late endosomes, and APP-positive organelles [76].

### 3.4. A Ubiquitin Switch in UL36p Sustains Processive Retrograde Transport

The processivity of dynein-mediated capsid transport is likely to be enhanced by the presence of dynactin and opposed kinesin motors (Section 3.1) [9,46,47] and possibly also elevated levels of Lis1 (Section 3.2) [48]. However, long-distance retrograde transport of capsids also requires UL36p ubiquitination at a conserved lysine residue close to the amino terminus of the protein (Figure 3) [77]. When this ubiquitination was prevented by mutation of the lysine to an arginine in the UL36p of PRV, the resulting UL36p-associated capsids no longer demonstrated sustained retrograde transport. Instead, they exhibited bidirectional motion along MTs with frequent stops and a PRV strain expressing the mutant UL36p was avirulent in a rodent intranasal infection model [77]. It is appealing to hypothesize that the ubiquitination state of UL36p directly controls its ability to bind or activate dynein/dynactin, however this remains to be demonstrated.

What factors control this ubiquitination? The responsible E3 ubiquitin ligase has yet to be identified but deubiquitination is performed by a conserved deubiquitinase (DUB) cysteine protease in the amino terminal portion of UL36p itself (Figure 3) [78,79,80]. As might be expected, mutations that inactivate this DUB (thus locking UL36p into the ubiquitinated, highly processive state) demonstrate efficient retrograde axonal transport when injected directly into the rodent CNS [77]. Surprisingly however, transmission of such mutants from epithelial cells into neurons was severely impaired, and UL36p DUB-defective PRV strains were unable to spread from peripheral epithelial tissues into neurons [77]. Thus, while inability to remove ubiquitin was compatible with neurotropism, it abolished neuroinvasion. The reason for this transmission defect from epithelia into neurons is unknown, but highly processive retrograde transport in polarized epithelial cells might misdirect virions to the epithelial apical surface [6] or accumulate them at the microtubule organizing center (MTOC), reducing the efficiency of their release in the vicinity of sensory neurons. Another unresolved question is how the UL36p DUB activity remains suppressed during trafficking in the axon, preserving UL36p in the ubiquitinated, processive retrograde trafficking state.

### 3.5. Retrograde Transport Functions Provided by the Inner Tegument Protein UL37p

The inner tegument protein UL37p (Figure 3) has been known for some time to contribute to retrograde traffic since its loss delays trafficking of PRV capsids to the nucleus even in non-polarized epithelial cells [81]. Most strikingly, mutation of a conserved surface-exposed region of UL37p termed R2 (Figure 3) abolishes the ability of PRV and HSV-1 strains carrying the mutant allele to invade the peripheral nervous system in vivo [82]. Virion entry, disassembly of outer tegument, and retention of UL36p/UL37p on the capsid are not affected by ablation of R2. Rather, capsids associated with R2-ablated mutant UL37p exhibit “ping pong” bidirectional movement along MTs instead of long-distance retrograde transport [82]. One possible explanation for these observations is that the UL37p-R2 region normally suppresses a kinesin motor activity present on the retrograde capsid (Section 3.1) [82]. Alternatively, R2 might be required to potentiate dynein/dynactin recruitment (or activation) by maintaining UL36p in its ubiquitinated “sustained retrograde” state (Section 3.4).

In addition to roles described above, UL37p could support retrograde traffic by providing a supplementary mechanism of dynein/dynactin recruitment. Although there is strong evidence that UL36p is the key effector recruiting dynein to the capsid [69,77,82], UL37p has been shown to bind dystonin/BPAG1 (bullous pemphigoid antigen 1) [83] in a yeast two-hybrid assay (Figure 3) [84]. Dystonin is a large protein with multiple isoforms that interacts with the actin cytoskeleton and with clathrin, and that plays roles in MT stability and Golgi organization [83,85]. BPAG1n4, a neuronal isoform, associates with the p150^Glued^ subunit of dynactin [66,86] and also with retrolinkin [87], a membrane protein enriched in neuronal endosomes. Together, these data suggest dystonin can connect membrane-embedded receptors on organellar cargo with the dynein/dynactin complex. Indeed, disrupting the interaction between dystonin and dynactin, or between dystonin and retrolinkin, causes defects in retrograde axonal transport of organelles and vesicles in uninfected neurons [86,87]. The importance of a UL37p/dystonin-dynein/dynactin recruitment pathway for alphaherpesvirus transport in neurons remains to be demonstrated, but shRNA-mediated depletion of dystonin in human fetal foreskin fibroblast 2 (HFFF2) cells delayed onset of HSV-1 gene expression following infection [88], consistent with an entry defect. Unexpectedly, however, the defect was not in dynein-mediated retrograde traffic of capsids from the cell periphery to the MTOC, but in anterograde movement away from the MTOC towards the cell nucleus. This is discussed in more detail in Section 4 below.

The HSV-1 UL37p protein has additional functions including a putative role in docking capsids to organelles during assembly [26,27] (Section 5.3) and a deamidase activity that acts upon retinoid-acid inducible gene-I (RIG-I) and cyclic GMP-AMP synthase (cGAS) to antagonize the innate immune response to HSV-1 infection [89,90]. However, the deamidase activity appears not to be conserved outside of the genus *Simplexvirus* [91] and an HSV-1 strain carrying an inactivating mutation in the UL37p deamidase active site is unimpaired in its ability to undergo retrograde axonal transport in primary cultured sensory neurons and in a mouse ocular infection model [91].

## 4. Climbing out of a Well: Trafficking from the MTOC to the Nucleus

After completing their long retrograde journey along the axon, alphaherpesvirus capsids enter the neuronal soma and reach the minus ends of MTs at the MT organizing center (MTOC) (Figure 3). Having devoted so much effort to retrograde traffic, capsids must now switch to anterograde transport to climb away from the MTOC and reach the cell nucleus. Since retrograde traffic is normally punctuated by short periods of anterograde motion [9,46], it would appear that kinesin motors are already present and available on the capsid surface, but how capsids switch from the processive retrograde state to one favoring anterograde transport is unknown. Another outstanding question is how anterograde-directed capsids choose the correct MTs to deliver them to the proximity of the nucleus, rather than to the periphery of the cell body or back down the axon.

One clue concerning this step comes from the finding that shRNA-mediated depletion of the UL37p-interacting protein dystonin (Figure 3) in HFFF2 cells led to a defect in anterograde traffic from the MTOC (Section 3.5) [88]. Loss of dystonin resulted in an approximately two-fold decrease in the numbers of capsids trafficking away from the MTOC, and a four-fold accumulation of capsids in the vicinity of the MTOC [88]. However, the role of dystonin and UL37p in this process remains unclear: there is no evidence that dystonin links cargo to kinesin motors [83] and it is not known whether the observed effects of dystonin depletion are mediated via UL37p. Dystonin silencing changes the appearance of the MT network in the vicinity of the nucleus even in uninfected HFFF2 cells [84], and in neurons loss of the neuronal isoform dystonin-a2 results in MT destabilization and the indirect disruption of motor-driven anterograde transport [85]. Cellular depletion of dystonin could therefore impact anterograde traffic of capsids from the MTOC to the nucleus by destabilizing the MTs that mediate it, rather than by affecting the recruitment of trafficking factors via UL37p. A PRV strain carrying inactivating mutations in the R2 surface in UL37p (Figure 3) (Section 3.5) and that is defective in long-range axonal retrograde transport shows normal entry into transformed epithelial cells, and into the soma of DRGs. This suggests that the R2 portion of UL37p is dispensable for capsid delivery to nuclei from the cell body surface and from the MTOC [82].

Upon reaching the nucleus, the inner tegument protein UL36p attaches the capsid to nucleoporins of the nuclear pore complexes (Figure 1) and the viral dsDNA genome is released into the nucleoplasm [12,92,93,94,95,96,97]. This is followed by the establishment of latency and maintenance of the viral dsDNA in the neuronal nucleus as a circular episome [5].

## 5. Anterograde Trafficking of Progeny Capsids and their Cytoplasmic Envelopment

### 5.1. The Emergence of Progeny Naked Capsids from the Nucleus and Their Trafficking in the Cytoplasm

**Figure 4:**
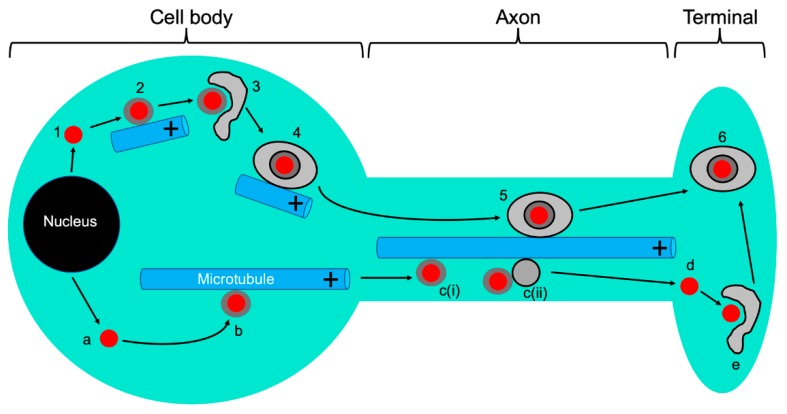
Alphaherpesvirus anterograde traffic in neurons. Capsids (red discs) are assembled and packaged with DNA in the nucleus, then emerge into the cytoplasm. Steps 1–6: married model. Naked capsids (1) acquire inner tegument and recruit kinesin to traffic along MTs (2) to their site of envelopment in the cell body (3). The resulting OEVs (4) then utilize MTs for delivery into and along the axon (5) eventually arriving at the nerve terminal for exocytosis and spread (6). Steps a–e: separate model. Naked capsids (a) acquire inner tegument and recruit kinesin to traffic along MTs (b) into the axon and along axonal MTs as naked capsids [c(i)]. In a variant of the separate model the naked capsid hitch-hikes on the surface of an anterograde trafficking carrier vesicle [c(ii)]. In both cases, the naked capsid moves down the axon separately from the lipids and proteins of its envelope. After arriving at the nerve terminal (d), the capsid is enveloped (e) to generate organelle-bound infectious virions indistinguishable from those delivered by the married model (6).

Following reactivation from latency, the viral genome is replicated and packaged into preassembled viral procapsids [1,12,15,98]. DNA-containing nucleocapsids then proceed to the inner nuclear membrane where they undergo primary envelopment and budding into the perinuclear space. Fusion with the outer nuclear membrane delivers naked capsids to the cytoplasm [99,100,101,102,103] (Figure 4). The inner tegument proteins UL36p and UL37p are recruited to the surface of the mature capsid shell early in this process, though whether this occurs in the nucleoplasm or following arrival of capsids into the cytoplasm is unclear for HSV-1 [12,95,104] and PRV [12,24,105,106]. There is, however, strong evidence that UL36p and UL37p are important for subsequent anterograde MT-directed traffic of capsids through the cytoplasm, from the nucleus and toward their site of secondary envelopment (Figure 4) [12,107]. Deletion of the UL37 or UL36 genes disrupts MT-directed anterograde HSV-1 capsid traffic in infected epithelial cells and mouse dorsal root ganglia (DRG): the resulting capsids demonstrate random, undirected diffusion in the cytoplasm [107,108]. UL36p is known to be essential to bind UL37p to the capsid (Section 2), so the simplest explanation for these data is UL36p is required to recruit UL37p, which in turn mediates kinesin recruitment. However, it is also possible that UL36p cooperates with UL37p to create a kinesin binding site [107,108]. Either model is consistent with biochemical studies showing that purified HSV-1 capsids bind kinesin-1 and kinesin-2 in vitro, but only if UL36p/UL37p are present and not obscured by outer tegument proteins [68]. Deletion of UL36p is also known to abolish the ability of HSV-1 capsids to undergo anterograde motility in an in vitro MT-dependent trafficking assay [109,110]. Interestingly, results obtained with PRV were slightly different: UL36p was essential for MT-directed anterograde trafficking of naked cytoplasmic PRV capsids but trafficking still occurred in the absence of UL37p, albeit with reduced kinetics [111]. This may suggest differences in the “distribution of effort” between UL36p and UL37p in kinesin recruitment by these two alphaherpesvirus.

### 5.2. Reorganization of Microtubules during Alphaherpesvirus Infection

Following emergence from the nucleus, the egressing alphaherpesvirus particle appears remarkably similar in structure to that responsible for initial infection, a non-enveloped naked capsid associated with the inner tegument proteins UL36p and UL37p (Figure 4). Both populations of capsid must also be capable of MT-directed anterograde traffic, from the MTOC to the nucleus (during entry), or away from the nucleus to the cell periphery or axon (during egress). Presumably there are molecular features of each particle, or the environment of the infected cell, that direct each capsid species to its correct destination. One way this could be achieved is by reorganization of the MT network during viral replication, and it has long been known that HSV-1 infection results in hyperacetylation, stabilization, and bundling of MTs in fibroblasts and epithelial cells [10,112,113,114]. Furthermore, during HSV-1 infection of epithelia and fibroblasts the MTOC is disrupted and MT growth instead originates from multiple new nucleating centers, including the TGN (trans Golgi network) distributed throughout the cytoplasm [113,114,115]. The HSV-1 serine/threonine kinase US3p plays an important role in MT acetylation and stabilization, in part by inactivating cellular GSK3β (glycogen synthase kinase-3) and de-repressing the activity of MT-stabilizing CLASPs (cytoplasmic linker-associated proteins), specialized +TIPs that control MT formation at the TGN [114]. CLASPs are required for HSV-1-induced MT stabilization and their depletion reduces infectious HSV-1 virion production and spread (see also Section 8) in primary normal human dermal fibroblasts (NHDFs) [114]. One of the most common and conserved sites of MT acetylation, the lysine-40 residue of α-tubulin, increases the life span of MT’s [116] and in neurons favors binding by kinesin-1/KIF5 and their utilization for transport of kinesin-1/KIF5-directed cargo [117]. In this way, post-translational modification of subsets of MTs, and assembly of novel infected cell-specific MT nucleating centers, could direct traffic of naked alphaherpesvirus capsids away from the nucleus, toward their sites of cytoplasmic envelopment and subsequently to the cell surface [114] (Section 8). However, disruption of the MTOC and generation of surrogate TGN-derived MT nucleating centers are not seen during PRV infection [115] and it is unclear whether HSV-1 and PRV manipulate the MTOC upon reactivation in neurons.

### 5.3. Coordination of MT-Directed Transport of Capsids with Cytoplasmic Envelopment

Anterograde transport through the cytoplasm delivers naked capsids to the surfaces of cytoplasmic organelles (Figure 4), within which are embedded the viral envelope proteins [118,119,120,121,122,123,124]. After docking with the organelle, budding of capsids into the lumen occurs in concert with recruitment of remaining outer tegument proteins [12,125,126] and acquisition of the mature viral envelope. Envelopment and ultimately scission of the envelope neck requires the coordinated activity of multiple viral proteins and the cellular ESCRT (endosomal sorting complex required for transport) apparatus [12,13]. Deletion of the UL36 or UL37 genes abolishes cytoplasmic envelopment of HSV-1 and PRV capsids [14,22,28,29,127,128], and interestingly mutation of two tryptophan motifs conserved in the UL36p proteins of all HSV-1 and HSV-2 isolates (W^1766^D^1767^ and W^1862^E^1863^ in HSV-1 strain 17^+^) (Figure 3) does not affect binding of UL36p or UL37p to capsids but inhibits envelopment and leads to accumulation of naked capsids in the vicinity of the MTOC [129]. These WD/WE “tryptophan-acidic” motifs resemble the bipartite signal that mediates binding of cargo to the carboxy-terminal tetratricopeptide repeats of KLCs (kinesin light chains) [130,131,132] and thus could be responsible for the ability of HSV-1 capsids with exposed inner tegument to bind kinesin-1/KIF5 in vitro [68]. It is possible that mutation of the UL36p WD/WE motifs disrupts kinesin recruitment to the inner tegument, impairs capsid anterograde traffic in the cytoplasm, and results in failure to deliver capsids to the envelopment site [129]. Alternatively, these conserved UL36p tryptophan residues could mediate conformational changes or intermolecular associations that more directly influence envelopment [129]. Although UL36p is not essential for capsid/membrane docking [133], the fidelity of capsid/organelle targeting is impaired by loss of UL36p [133] and in its absence capsids aggregate both in the cytoplasm and on the organellar surface [28,133]. Inactivation of UL36p also diminishes the ability of HSV-1 capsids to recruit the cellular ESCRT machinery [13,134].

Like UL36p, UL37p is essential for HSV-1 and PRV capsid envelopment [12,22,29,128]. As for UL36p, this could be because UL37p is required for MT-directed anterograde trafficking of capsids to their envelopment site, though this trafficking defect is less pronounced in PRV [111] than in HSV-1 [107,108] (Section 5.1). The structural similarity between the amino terminal portion of UL37p and cellular MTCs [26,27] (Section 2) also suggests the possibility that UL37p might dock capsids to the surface of their envelopment organelle, possibly via binding to the alphaherpesvirus envelope protein heterodimer gK/UL20p [135,136]. This would explain why in the absence of UL36p (and thus UL37p) the fidelity of capsid/organellar targeting is impaired [133]. Finally, as described earlier (Section 4), UL37p has been shown to associate with dystonin/BPAG1 [83] in a yeast two-hybrid assay (Figure 3) [84]. Dystonin depletion inhibited anterograde trafficking of HSV-1 capsids through the cytoplasm during egress in HFFF2 cells [84] (as also seen during capsid anterograde traffic from the MTOC to the nucleus during entry, Section 4), but the role of dystonin and its relationship with UL37p in this anterograde transport remains unknown.

### 5.4. MT-Directed Transport of Enveloping Capsids Is Arrested Until Envelopment Is Complete

The envelopment of naked cytoplasmic capsids to generate organelle-enclosed enveloped virions (OEVs) (Figure 4) is an important transition in the trafficking problem for alphaherpesviruses. Naked capsids are relatively small, ~125 nm diameter rigid proteinaceous particles. In contrast, OEVs are at least ~200 nm in diameter [134,137,138] and possibly much larger if multiple enveloped virions accumulate within the lumen of the carrier organelle. These structures will presumably require greater force to move them along MTs than that required for capsids, and their bounding lipid membrane creates a far more flexible surface for motor attachment than the rigid capsid shell. Members of the kinesin family differ considerably in their force generation and the efficiency with which they operate in teams to move large and small cargo [139,140,141], it is therefore possible that different anterograde motors are adopted by naked capsids and OEVs during their transport (discussed in detail in Section 7 below).

**Figure 5:**
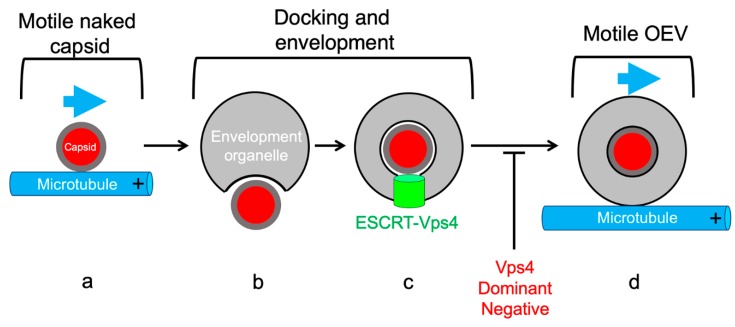
The alphaherpesvirus envelopment intermediate is non-motile. (**a**) Naked capsids (red disc) with inner tegument (grey layer) are capable of anterograde traffic along MTs (blue arrow). (**b**) Following delivery to the envelopment organelle capsids dock and bud into the lumen to assemble infectious enveloped virions. (**c**) The ESCRT apparatus and the Vps4 ATPase (green cylinder) catalyzes scission at the bud neck to pinch the enveloped virus into the organellar lumen. This envelopment intermediate is non-motile until scission is completed, and can be irreversibly trapped using a dominant negative allele of Vps4 (red text). (**d**) If scission is successful, the resulting organelle, containing a lumenal enveloped alphaherpesvirus particles, acquires the ability to traffic along MTs.

Since HSV-1 [137,142] and PRV [142] utilize the ESCRT apparatus (Section 5.3) to complete envelopment [13], the process can be arrested by expression of a dominant negative allele of Vps4 (vacuolar protein sorting 4), a cellular AAA ATPase that completes ESCRT-mediated scission, and envelope sealing (Figure 5) [143,144,145]. The resulting arrested intermediates, capsids partially but not completely enclosed by membrane, resemble an egg sitting in an eggcup [134,137]. Interestingly, we found that for both HSV-1 and PRV these trapped assembly intermediates were incapable of trafficking along MTs in an in vitro system (Figure 5), even though MT-directed trafficking of naked capsids and fully mature OEVs was normal [142]. Inhibition of motility was not an artifact of arresting envelopment with the dominant negative allele of Vps4, because the rare and short-lived population of partially enveloped capsids undergoing normal envelopment in the presence of wild-type Vps4 were also unable to move along MTs until envelopment had been completed [142]. Thus, while “precursor” naked alphaherpesvirus capsids and “product” OEVs can traffic along MTs, ESCRT-associated envelopment intermediates cannot (Figure 5). Although this mechanism remains to be demonstrated in vivo, we have hypothesized that this reflects an assembly checkpoint: once naked capsids have trafficked to and entered the envelopment pathway, any further MT-dependent trafficking is prevented until OEVs have been correctly assembled and the cargo of infectious virions is ready for export [142].

## 6. Anterograde Trafficking Down the Axon: A Multitude of Trafficking Particles and of Models to Account for Them

### 6.1. The “Married” and “Separate” Models

Alphaherpesvirus particles in the neuronal cell body must eventually enter the axon and travel back along it to the nerve terminal by MT-directed anterograde transport. Mature infectious viral particles are then released to infect and undergo productive replication in adjacent epithelial cells [2] (Figure 4). Only a small subset of the cytoplasmic viral particles traffic from the cell body into and down the axon in cultured neurons, though for reasons that are unclear, HSV-1 delivers considerably fewer virions to the axon than does PRV. Our understanding of the molecular details of alphaherpesvirus anterograde axonal transport is complicated by fundamental questions concerning the structure of the trafficking viral particle. Data from a number of laboratories has led to the development of two models, summarized in Figure 4, whose fundamental differences arise from the location at which capsids undergo cytoplasmic envelopment in neurons [146,147,148]. In the “married” model, capsids emerge from the cell nucleus then undergo envelopment in the neuronal cell body to generate OEVs that enter and traffic down the axon. Alternatively, in the “separate” model, naked capsids travel from the cell body into the axon, along axons, and eventually encounter their envelopment organelles at a distal site such as the nerve cell terminal (Figure 4). Distinguishing between these two models is important because they have profound implications for the molecular mechanisms of alphaherpesvirus assembly, transport, and motor recruitment. There is general consensus in favor of the married model for PRV trafficking, but for HSV-1 the mechanism remains contentious and a number of technical issues have complicated interpretation. For example, infection of dispersed cultured neurons can occur asynchronously over many hours. This means that late in infection, newly entering retrograde-trafficking naked capsids may exist in the axon during times when one would expect progeny virions to be leaving the cell body. The use of microfluidic devices or Campenot chambers that physically separate axons from cell bodies has helped to address this problem [149]. Additionally, detection of viral capsid proteins by immunofluorescence can be confounded by limited accessibility of antibody to the antigen in viral particles or transport vesicles. Testing for the colocalization of fluorescent fusions made to capsid and envelope proteins is an attractive alternative to demonstrate the presence of married particles, however such an approach requires the construction and expression of recombinant proteins and viruses. Many of these issues have been discussed in detail elsewhere [2,44,138,146,147,149,150].

In infected explanted human fetal DRGs, HSV-1 capsids were found to be transported along axons independently of tegument and envelope proteins, consistent with the separate model, and capsids were found partially and fully enveloped in axonal varicosities and growth cones, suggesting these were the sites of envelopment for at least some HSV-1 capsids [151,152,153]. Similarly, HSV-1 capsids were reported to traffic along axons via the separate mechanism in rat DRGs, rat TGs, embryonic rat hippocampal neurons, and cultured human SK-N-SH neuroblastoma cells [138,154,155]. Naked capsids were also observed trafficking in the axons of murine retinal ganglia following HSV-1 intraocular infection [156]. In contrast, studies using neurons derived from mouse DRGs led to the conclusion that HSV-1 capsid envelopment must be completed in the soma as a prerequisite for entry into the axon, as required by the married model [108]. Similar conclusions were reached for HSV-1 transport in differentiated mouse CAD cells [Cath.a-derived, a derivative of a catecholaminergic central nervous system cell line [157,158]] and in rat and chick DRGs [44,159]. In rat SCGs, one study found only completely assembled enveloped virions (OEVs) within axons upon infection with HSV-1, HSV-2, or PRV [150], but another found a mixture of OEVs and naked capsids in a 3:1 ratio [160]. To reconcile such findings it has been suggested that HSV-1 may have the capacity to utilize *both* the separate and married transport mechanisms, with varying ratio of naked axonal capsids and OEVs, depending upon the origin of the neurons being studied and how transport is assayed [160,161,162] (see also Section 7.4).

### 6.2. Trafficking of Viral Glycoproteins in the Absence of the Viral Capsid

An obligate aspect of the separate model is that the lipid bilayer to be utilized for envelopment, viral envelope proteins embedded within it, and possibly outer tegument proteins all be delivered to the nerve terminal by a separate parallel pathway to that used for anterograde MT-directed transport of capsids. In HSV-1 infected human fetal DRGs, where several studies have been published in support of the separate pathway [151,152,153,163], an immunogold analysis suggested that outer tegument and envelope proteins were transported down axons in pleiomorphic tubulovesicular structures and mid to large dense-cored vesicles (DCVs) [152]. These carriers frequently labeled for the TGN antigen TGN46 [152] and contained markers of the neuronal exocytic pathway including Rab3A, GAP43 (growth-associated protein 43), and SNAP25 (synaptosome-associated protein 25). Trafficking-organelles that labeled for outer tegument proteins were also immuno-reactive with antibodies against kinesin-1/KIF5, consistent with the finding that in uninfected neurons, SNAP25 carrier vesicles are transported by KIF5 motors [141], though Rab3A-positive DCVs are usually associated with kinesin-3/KIF1A [141,164]. Interestingly, antibodies to kinesin-1/KIF5 densely labeled enveloped capsids at varicosities and growth cones, as well as extracellular virions adjacent to axons [152], suggesting that this motor remains in close association with the HSV-1 particle after envelopment and exocytosis.

Whereas the separate model requires that viral capsids and envelope glycoproteins undergo independent anterograde transport down the axon, the converse is not true: glycoprotein trafficking along the axon can occur independently of capsids for reasons unrelated to the separate model. A feature of tissue culture replication common to all alphaherpesviruses examined is the production of L (Light)-particles, similar in size to mature virions (though more heterogeneous) and consisting of lipid envelope-like structures with membrane-embedded envelope proteins and tegument, but lacking capsids [12,165,166,167,168]. These non-infectious particles are thought to arise when capsids fail to engage with the envelopment pathway in the cytoplasm, but where envelopment nevertheless still occurs [12], presumably driven by tegument, envelope proteins and the ESCRT-apparatus [12,13]. This generates organelles containing enveloped particles with envelope proteins and tegument but lacking capsids [169]. Consistent with the married mechanism of transport for PRV, PRV L-particles have been detected in axons in vitro and in vivo [159,170,171,172]. In contrast, in embryonic rat hippocampal neurons, where HSV-1 capsids have been proposed to utilize the separate pathway for assembly and transport [138], HSV-1 L-particle assembly has been reported to occur at the axon terminus [138,173]. As detailed below (Section 7.1), there is evidence that additional pathways exist to transport viral structural components along axons.

## 7. The Virally Encoded Membrane Proteins gE, gI, and US9p Play Key Roles in Axonal Trafficking and Spread of Alphaherpesvirus Particles

### 7.1. The Virally Encoded Membrane Proteins gE, gI, and US9p: An Overview

Three virally encoded membrane proteins play important roles in the anterograde traffic of HSV-1 and PRV (Figure 2) [2,16,174,175,176,177,178,179]. US9p is a small (90 amino acid long in HSV-1 strain 17) non-glycosylated lipid raft-associated type II membrane protein, with its carboxy terminal ~20 amino acids embedded in the lipid bilayer and the amino terminus of the protein directed towards the cytoplasm or the viral tegument (Figure 2) [180,181,182]. Glycoproteins E and I (gE and gI) are type I membrane proteins with 300- to 400-amino-acid long extracellular domains and 90- to 110- amino acid cytoplasmic tails that appear to always function in the context of a gE/gI heterodimer. In polarized epithelial cells in culture, gE/gI are required for the efficient sorting of HSV-1 virions to lateral surfaces and cell junctions, and loss of gE/gI results in HSV-1 mis-sorting to the apical surface and poor cell-cell spread between cultured polarized epithelial cells and in epithelial tissues [16,183].

In HSV-1, gE/gI and US9p are important for anterograde spread within the nervous system in a cooperative manner [184,185,186,187] with some degree of redundancy: loss of US9p reduces delivery of HSV-1 particles to distal axons by about 50% [161,188], but simultaneous loss of US9p and gE/gI abolishes delivery to axons almost completely [189]. In contrast, PRV strains with the US9 gene deleted are completely defective for axonal sorting of viral particles in mature cultured neurons and exhibit no anterograde spread of infection [170,182,190,191]. The absence of US9p similarly results in failure of most PRV envelope proteins to enter the axon [172,182,190]. This may be because most axonal PRV envelope proteins are components of OEVs (organelles carrying enveloped infectious PRV particles or L-particles, Section 6.2) [170], alternatively viral glycoproteins might traffic down the axon in transport vesicles utilizing the same US9p-dependent transport mechanism as OEVS [170,182]. Nevertheless, anterograde MT-directed transport of at least one PRV encoded glycoprotein, gM, is not US9p-dependent [172]. HSV-1 glycoproteins show variable dependence upon gE/gI and US9p for delivery to the axon, as detailed below (Section 7.3).

### 7.2. Molecular Roles for gE/gI and US9p in Anterograde Transport of Enveloped Virions (the Married Model)

What are the molecular functions of gE/gI and US9p in the context of the married and separate models for alphaherpesvirus anterograde transport along MTs? In the case of PRV, which is generally agreed to follow the married model, an appealing possibility is that gE/gI and US9p serve to recruit kinesin motors to the surface of trafficking OEVs. PRV US9p copurifies with the kinesin-3 motor KIF1A in extracts of PRV infected PC12 cells [192] and US9p is known to be associated with trafficking anterograde virions [170,192]. Alanine substitution of tyrosine residues Y^49^ and Y^50^ in the cytoplasmic tail of US9p generates a US9p mutant protein that is unable to coimmunoprecipitate with KIF1A [192], results in greatly diminished numbers of capsid-associated PRV particles in the axons of cultured SCG’s [170] and fails to support PRV anterograde spread in a rat eye infection model [181]. The gE/gI heterodimer does not copurify with US9p/KIF1A [192,193] but it is required for efficient formation or maintenance of the US9p/KIF1A complex [193], and gE interacts with a population of US9p that is not KIF1A-associated [193]. Together, these data are consistent with the possibility that US9p is a membrane receptor that directly recruits KIF1A to the surface of OEVs for anterograde traffic along the axon, and that gE/gI indirectly modulates US9p to stimulate or stabilize this association [192,193].

More recent findings have complicated this interpretation. Deletion of the US9 gene clearly results in fewer PRV particles present in axons, nevertheless the kinetics of anterograde transport of those few axonal US9p-null PRV particles were indistinguishable from wild type [194]. Similar data were reported for HSV-1, where simultaneous loss of US9p and gE/gI led to severely diminished numbers of trafficking axonal particles, but those in the axon exhibited normal rates of anterograde traffic [161,189]. One possible explanation is that the reduced numbers of axonal viral particles seen in ΔUS9 PRV and ΔUS9/ΔgE/ΔgI HSV-1 strains is due to aberrant trafficking of viral particles within the neuronal cell body, or a failure to efficiently sort virions from the cell body into the axon itself [194]. Delivery into the axon requires that alphaherpesviruses traverse the axon initial segment (AIS), a region of the axon close to the cell body that acts as a barrier to free diffusion of cytoplasmic contents and plasma membrane proteins and lipids, and thus helps maintain axonal identity (reviewed in [195]). AIS MTs are arranged into widely spaced high density string-like bundles termed fascicles and are composed of tubulin rich in post-translational modifications including acetylation, detyrosination, and polyglutamylation [195]. This unique MT organization depends in part upon ankyrin G, which connects MT-bound EB1 and EB3 (Section 3.3) with the actin/spectrin submembrane scaffold. Normal cellular transport vesicles destined for the axon enter and traverse the AIS with no apparent delay, however vesicles carrying somatodendritic proteins are either excluded by the AIS or enter the AIS but stall and reverse direction [195]. The mechanism by which the AIS discriminates between axonal and non-axonal cargo is unclear, but it has been proposed that the environment of the AIS imposes a high viscous drag [195,196]. In this model, only motor-cargo complexes capable of a sufficiently high rate of trafficking can push through the AIS into the axon [196]. It is possible that US9p-mediated recruitment of KIF1A, in cooperation with gE/gI, generates trafficking viral particles that are able to achieve the velocities required to penetrate the AIS.

If US9p-KIF1A mediates transport of PRV and HSV-1 alphaherpesvirus particles within the cell body and are responsible for delivery of virions into the axon, are other motors and motor-receptors utilized for subsequent axonal trafficking? In cultured differentiated mouse CAD cells anterograde-trafficking HSV-1 particles in the axon frequently colocalized with GAP43 and SNAP25, cargo commonly transported by kinesin-1/KIF5 [197] (these same cellular proteins were found to be associated with the envelopment organelles utilized by HSV-1 capsids at nerve terminals in human fetal DRGs, Section 6.2 [152]). Similarly, a fluorescently tagged allele of KIF5C showed substantial colocalization with axonal HSV-1 particles, whereas a fluorescent KIF1A did not [197]. Furthermore, silencing of the three kinesin-1 subtypes KIF5A, -5B, and -5C, or KLC1 and KLC2 (kinesin light chains-1 and -2) [140] inhibited most HSV-1 transport in axons, while silencing of KIF1A had little effect [197]. How might kinesin-1/KIF5 be recruited to OEVs to transport enveloped HSV-1 particles down the axon? An early study using a squid axon model demonstrated that anterograde-trafficking HSV-1 particles contained APP (amyloid precursor protein) [198], a putative kinesin-1 receptor [199]. Furthermore, KIF5C-associated anterograde-directed HSV-1 particles colocalized with APP in CAD axons [197]. Nevertheless, interpretation of this finding is complicated by the fact that the role of APP in kinesin recruitment is not entirely clear [141,200]. Another possible kinesin-1 receptor is US9p itself, which for HSV-1 has been reported to directly interact with the carboxy-terminal tail of KIF5B [201]. However, if US9p is essential for kinesin-1 recruitment, then loss of US9p should disrupt the rate of trafficking of axonal HSV-1 particles, which does not seem to be the case [161,189].

An additional role for US9p and gE/gI within the context of the married model (Figure 4), that is not necessarily exclusive of a function in motor recruitment, is that these proteins participate in the envelopment of capsids in the cell body. An HSV-1 strain simultaneously deleted for gE (therefore functionally null for the gE/gI heterodimer) and US9 accumulated larger numbers of partially enveloped capsids than did wild type control viruses in the cell body of rat embryonic SCG neurons, mouse CAD neurons, and human SK-N-SH neurons [161]. In the married model, an envelopment defect in the cell body would of course lead to fewer enveloped particles available for transport (Figure 4), explaining why diminished numbers of enveloped virions are seen in axons when gE/gI and US9p are absent. This model is consistent with the finding that capsid envelopment is a prerequisite for delivery of HSV-1 particles to the axon in adult mouse DRGs [108] and with our own in vitro data showing that partially enveloped HSV-1 and PRV capsids are blocked in their ability to traffic along MTs (Figure 5) [142] (Section 5.4). Other studies have proposed a role for US9p in HSV-1 capsid envelopment in human and rat DRGs. In that case, the envelopment defect was interpreted to be in neuronal growth cones and varicosities, following axonal transport of naked capsids to those locations in accord with the separate model (Figure 4) [202].

### 7.3. Molecular Roles for gE/gI and US9p in Anterograde Transport of Naked Capsids and Viral Glycoproteins (the Separate Model)

Within the context of the separate model for HSV-1 assembly and transport (Figure 4), how can we explain the observation that loss of gE/gI and US9p impacts the MT-dependent trafficking of envelope glycoproteins and of naked HSV-1 capsids (Section 6)? Compared to wild type controls, HSV-1 mutants lacking gE or gI (thus in both cases functionally null for the gE/gI heterodimer) or the US9 gene showed ~50%–75% fewer naked capsids and viral glycoprotein-associated structures entering the axons of human SK-N-SH neurons, though transport of capsids appeared more sensitive to the deletions than did transport of glycoproteins [188]. Loss of US9p similarly reduced the numbers of trafficking naked HSV-1 capsids by ~50% in axons of human and rat DRGs [202] and essentially abolished it in axons of murine retinal ganglia [156], with more modest effects upon viral glycoprotein trafficking [156,188].

It is conceptually straightforward to envision how the membrane-embedded proteins gE/gI and US9p might gather viral envelope glycoproteins into carrier vesicles, sort them into the axon, and/or recruit kinesin motors to the vesicle to support MT-directed transport down the axon. It is considerably more difficult to understand how these membrane proteins could play roles in the trafficking of *naked* capsids through the AIS (Section 7.2) or down the axon [161]. One hypothesis is that gE/gI/US9p provide “loading” sites on the cytoplasmic surfaces of cell body organelles that recruit naked capsids, attach them to motors, and direct them to axonal MTs for subsequent transport [188,189]. However, the details of such a mechanism remain unclear. Further complicating matters, US9p (but not other virally encoded membrane proteins) has been reported to colocalize with trafficking naked HSV-1 capsids in the axon [156,188]. Perhaps the “naked’ trafficking capsids postulated by the separate model are in fact docked to the cytoplasmic face of an anterograde-trafficking vesicle carrier that contains membrane-anchored US9p and has the capacity to recruit motors (Figure 4) [188]. This “hitch-hiking” capsid would ride the vesicle to the nerve terminal then presumably depart from its carrier to engage the envelopment machinery (Figure 4). Interestingly, we observed vesicle-docked but non-enveloped HSV-1 capsids bound to MTs in our in vitro system that reconstitutes MT-mediated transport of HSV-1 [109,110]. Membrane-associated but non-enveloped capsids have also been observed in axons for both HSV-1 and PRV [2,151], but US9p-positive HSV-1 capsids did not appear to be membrane-associated in the axons of mouse retinal ganglia [156].

### 7.4. Summary of Roles for gE/gI and US9p in Anterograde Transport of Alphaherpesviruses in the Axon

Given the complexity of the literature in this area, we believe that it would be helpful to attempt to derive a consensus model for the roles of gE/gI/US9p in alphaherpesvirus anterograde transport in neurons, consistent with most published data (Figure 4). In the neuronal cell body, PRV capsids, and in some neurons at least a subset of HSV-1 capsids, undergo envelopment to generate the OEV. gE/gI and US9p in the bounding organellar membrane facilitate this envelopment then cooperate to recruit KIF1A, which delivers the organelle and its cargo of enveloped virions through the AIS and into the axon. Prior to, or upon arrival in the axon, the HSV-1 OEV binds kinesin-1 motors such as KIF5C by a process independent of gE/gI/US9p, and the KIF1A motor is lost or rendered undetectable [197]. In the case of PRV, the OEVs retain their KIF1A motors during anterograde transport down the axon [192] and may utilize them for traffic, but do not absolutely require KIF1A (or gE/gI/US9p) to be present for efficient anterograde transport to occur.

In some neurons, a subset of HSV-1 capsids bypass envelopment in the soma and instead traffic into the axon by a gE/gI/US9p-dependent mechanism that remains to be characterized. It is possible that the ability of naked capsids to enter the axons of some neurons but not others reflects differences in the filtering properties of the AIS (Section 7.2) that arise as a result of neuronal origin or culture conditions. Capsids might dock to the surface of a carrier vesicle which recruits kinesin motors via membrane-associated receptors in a conventional manner or alternatively motors bind directly to the capsid surface (Figure 4), possibly via the same inner tegument UL36p/UL37p machinery (Figure 3) used to support anterograde cytoplasmic trafficking (Section 5.1). Kinesin-1/KIF5 is an attractive candidate for axonal transport of these capsids since it has been demonstrated to bind naked HSV-1 capsids in vitro [68].

### 7.5. Motor Choice and Additional Viral Candidates for Kinesin Recruitment

The reasons why alphaherpesviruses might prefer one or another motor for transport within the cell body and axon remain unknown. KIF1A is one of the fastest anterograde motors [140,141] and in neurons transports synaptic vesicle precursors that contain synaptophysin, synaptotagmin, and Rab3A [140,141]. Although KIF1A exists as a monomer [141], it is highly processive when multiple copies of the motor cooperate in teams [139]. Nevertheless, recombinant KIF1A molecules designed to be obligate monomers demonstrate poorer force generation than dimeric kinesins [139]. Kinesin-1/KIF5 is dimeric, with the capacity to move high-load cargo [139] including organelles such as mitochondria, lysosomes, and synaptic vesicle precursors along axons [140,141] and ~1000S mRNA-containing ribonucleoprotein granules within dendrites [141,203]. The KIF5 motor domain also preferentially recognizes MTs in the proximal part of the axon, positioning it to meet and transport cargo arriving from the neuronal cell body [204]. Finally, as described in Section 7.2, combinations of cargo and kinesin motor that result in high rates of transport tend to be favorable for traversal of the AIS and entry into the axon [196].

Additional alphaherpesvirus-encoded proteins have been proposed to associate with kinesins, but their roles in egress remain to be established. The HSV-1 outer tegument protein US11p, a dsRNA-binding protein involved in translational regulation [205], interacts with kinesin-1/KIF5B [206] and with the KLC-related protein PAT1 [207] but there is no US11 homologue in the *Varicelloviruses* PRV and VZV. Moreover, HSV-1 capsids lacking US11p (and other outer tegument proteins) are able to bind kinesin-1/KIF5 in vitro, and outer tegument proteins inhibit this binding [68]. The UL56 gene product bears some similarity to US9p in that it is a type II membrane protein that partitions into lipid rafts and binds kinesin-3/KIF1A in yeast two-hybrid and GST pull-down assays [208]. UL56p is largely dispensable for replication in tissue culture but facilitates transport of HSV-2 out of cultured epithelial cells [209] and is important for virulence in a tree shrew model [210], however a role in MT-directed traffic remains to be established. Finally, the US3p kinase, known to affect MT stabilization and formation of secondary MTOCs in HSV-1-infected fibroblasts [114] (Section 5.2), has been shown to directly phosphorylate the kinesin-2 motor KIF3A, suppressing cell surface expression of the major histocompatibility complex class I-like CD1d molecule to facilitate immune evasion from natural killer T cells [211]. These studies were performed in HeLa and 293T cells but raise the possibility that US3p or other virally encoded kinases might reprogram cellular motors to control alphaherpesvirus egress in neurons. Interestingly, along with UL36p and UL37p the US3p kinase is one of the tegument components known to remain associated with the capsid during entry into neurons, and to accompany the capsid during its retrograde journey along the axon (Section 3.3) [44,59,60,61,62].

## 8. Final Steps: Exocytosis of Enveloped Virions at the Nerve Terminal

The final step in viral anterograde transport within neurons is delivery of the OEV to the cell periphery and fusion between the bounding organellar and plasma membranes, releasing alphaherpesvirus particles to infect adjacent epithelial cells. In HSV-1 infected human fetal DRGs, OEVs are associated with the cellular proteins Rab3A, SNAP25, and GAP43 [151,152], suggesting that HSV-1 particles pass through the same pathways used for delivery of synaptic vesicle proteins to the axon terminus, and for the regulated exocytosis of neurotransmitters and hormones [11,152]. In infected PK15 epithelial cells, PRV virions are delivered to the cell surface in Rab6A, Rab8A, and Rab11A-associated organelles, a similar Rab profile to that seen for vesicles mediating constitutive traffic of viral glycoproteins to the plasma membrane [212,213]. In these epithelial cells, MTs appear to direct PRV-carrying vesicles all the way to the cell surface, since most exocytic events occur near sites on the plasma membrane that label with fluorescently tagged LL5β [213], a PIP3-binding protein that connects CLASPs (Section 5.2) at the plus-end of MTs to the plasma membrane [75]. Depletion of CLASP reduces the efficiency of HSV-1 spread [114], consistent with a role for CLASP/LL5β in this final exocytic step for HSV-1 too, at least in NHDF cells. In most cases, each individual exocytic event results in delivery of a single PRV virion from the surface of PK15 cells [212,213]. It is unclear how mechanistically similar this is to release from neuronal cells, but spread of HSV-1 and PRV from cultured rat SCGs to adjacent epithelial cells is similarly thought to involve single particles [214].

## 9. Conclusions

The neurotropic alphaherpesviruses have evolved the ability to accomplish efficient retrograde and anterograde transport along neuronal MTs at different stages of their infectious cycle. A substantial body of work from many laboratories has led to an understanding of some of the molecular mechanisms by which these viruses coopt microtubules and molecular motors, but fundamental questions remain. Answering these questions will have great significance for understanding and controlling alphaherpesvirus pathogenesis, and in their development as safe gene delivery vectors for the nervous system.
